# Landscape structure and population density affect intraspecific aggression in beavers

**DOI:** 10.1002/ece3.6980

**Published:** 2020-11-26

**Authors:** Martin Mayer, Clàudia Aparicio Estalella, Steve K. Windels, Frank N. Rosell

**Affiliations:** ^1^ Faculty of Technology, Natural Sciences, and Maritime Sciences Department of Natural Sciences and Environmental Health University of South‐Eastern Norway Bø i Vestfold and Telemark Norway; ^2^ Department of Bioscience Aarhus University Aarhus Denmark; ^3^ Faculty of Biology University of Barcelona Barcelona Spain; ^4^ Voyageurs National Park International Falls MN USA

**Keywords:** *Castor canadensis*, *Castor fiber*, intraspecific aggression, monogamy, tail scars, territoriality

## Abstract

Intraspecific competition plays an important role for territory acquisition and occupancy, in turn affecting individual fitness. Thus, understanding the drivers of intraspecific aggression can increase our understanding of population dynamics. Here, we investigated intraspecific aggression in Eurasian (*Castor fiber*) and North American (*Castor canadensis*) beavers that are both monogamous, territorial mammals. Combined, we examined tail scars from >1,000 beavers (>2,000 capture events) as part of two long‐term studies in Norway and the USA. We investigated the influence of landscape structure, population density, sex, age, and (for Eurasian beavers only) social status and group size on the number of tail scars caused by conspecifics. The number of tail scars was affected by population density in well‐connected landscape types (large lakes and rivers), but not in more isolated areas (ponds), where individuals generally had fewer tail scars. Further, the relationship of population density was not linear. In the North American beaver population occurring in large lakes, intraspecific aggression increased with population density. Conversely, in the saturated Eurasian beaver population, intraspecific aggression was in a negative relationship with population density (except at the highest densities), likely due to inverse density‐dependent intruder pressure via dispersers. Our findings emphasize that population density can affect intraspecific aggression depending on landscape structure, which might have important consequences for local patterns of dispersal, mate change, and territory occupancy, all of which can affect population dynamics.

## Graphical abstract

We investigated intraspecific aggression in beavers using tail scars from >1,000 individuals. The number of tail scars was affected by population density in well‐connected landscape types (large lakes and rivers), but not in more isolated areas (ponds), where individuals generally had fewer tail scars. Our findings emphasize that population density can affect intraspecific aggression depending on landscape structure, which might have important consequences for local patterns of dispersal, mate change, and territory occupancy, all of which can affect population dynamics.

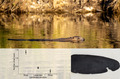

## 1 INTRODUCTION

Animals engage in agonistic behaviors for different reasons, for example, to gain access to food, territories or mating partners, to reinforce their social status within a group, or to fight off predators (Mirville et al., 2020; Morgan & Fine, 2020; Nussbaum et al., 2016). Variation in intraspecific aggression can be caused by various ecological factors and can have important ecological functions and consequences. For example, reduced intraspecific aggression might contribute to the success of invasive species (Holway et al., 1998; Krushelnycky et al., 2010), whereas increased aggression might be an adaptation of wildlife to urbanization or a result of higher population densities (Baxter‐Gilbert & Whiting, 2019; Parker & Nilon, 2008). More generally, density dependence plays an important role in intraspecific aggression, which can affect survival as shown in gray wolves (*Canis lupus*) (Cubaynes et al., 2014). Population density can be influenced by landscape structure, for example, via altered resource availability due to fragmentation (Nupp & Swihart, 1996). Further, landscape structure itself can affect aggressive encounters by facilitating or inhibiting connectivity between habitat patches (Allen et al., 2014; Verbeylen et al., 2009). Thus, understanding the combined effects of landscape structure and population density is valuable to improve our understanding of intraspecific aggression and its consequences for population dynamics.

Intraspecific agonistic behaviors are often the consequence of territory defense (Krebs, 1971; Piper et al., 2015), which allows individuals to obtain exclusive access to food, mating partners, and other resources (Wyatt, 2014). The defense of territories can lead to spatially structured populations, which regulates the number of territory holders, and thus population density (López‐Sepulcre & Kokko, 2005). Apart from territoriality, population structure is affected by landscape features, because they can affect dispersal and migration, both processes influencing gene flow (Manel et al., 2003). Although it is well known that landscape features can affect population structure, habitat selection, and movement behavior (Funk et al., 2005; Lendrum et al., 2012; Romero et al., 2009), little is known about the role of landscape structure on intraspecific aggression.

**FIGURE 1:**
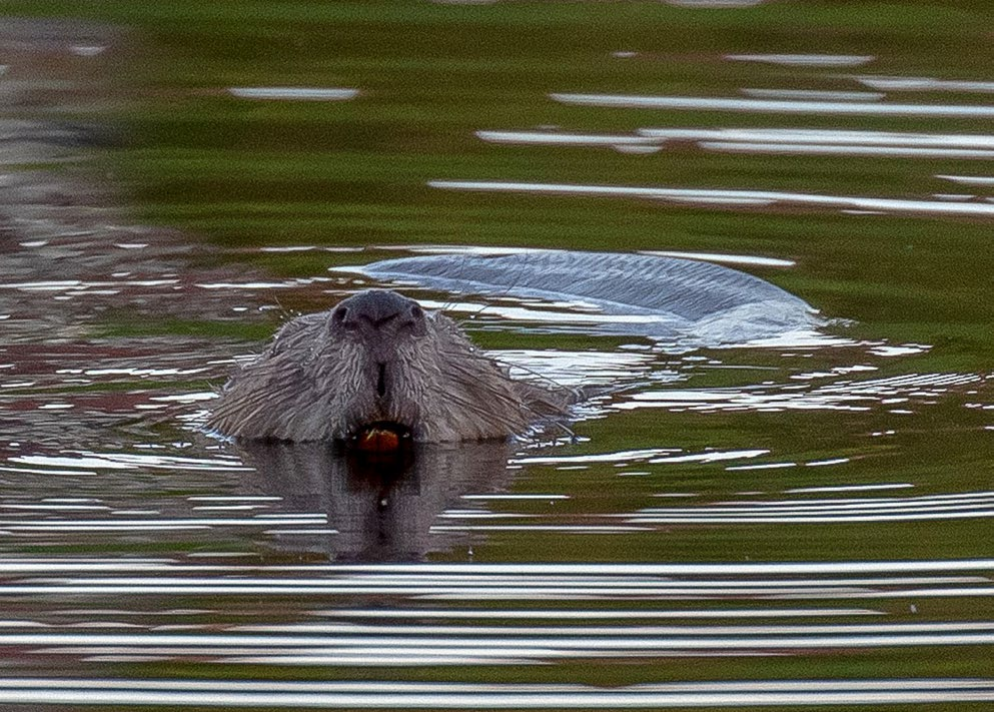
A Eurasian beaver (*Castor fiber*) in a river in South‐eastern Norway having his head lifted to sniff. Picture: Frank Rosell

Both the Eurasian beaver (*Castor fiber*; Figure 1) and the North American beaver (*Castor canadensis*) are highly territorial mammals (Hodgdon & Lancia, 1983; Hohwieler et al., 2018; Müller‐Schwarze & Heckman, 1980), making them an optimal model to study intraspecific aggression. They are obligate monogamous, live in family groups, and are very similar in morphology and ecology (Baker & Hill, 2003; Wilsson, 1971). Family groups comprise of the male and female territory owner and their offspring; the kits of the year, yearlings, and sexually mature (≥2 years old) nonbreeding family members (hereafter “subordinates”). Kits are born in spring and emerge from the lodge mid‐summer (Baker & Hill, 2003; Parker & Rosell, 2001). Subordinates typically disperse during spring at the age of two (Hartman, 1997; Sun et al., 2000), but can delay dispersal until the age of seven in saturated populations (Mayer et al., 2017; Mayer Zedrosser et al., 2017c). Individuals conduct extra‐territorial forays before dispersal, possibly to detect available territories (Hartman, 1997; Havens, 2006; Mayer, et al., 2017; Mayer Zedrosser et al., 2017b). Once established, territory occupancy is advertised through scent marking (Rosell et al., 1998).

Aggressive encounters are not rare, and beavers are frequently injured during intraspecific competition, with scars accumulating with increasing age (Crawford et al., 2015; Mayer et al., 2020). Beavers rarely appear to die outright from such wounds, but some cases of mortality have been recorded as a result of intraspecific aggression (DeStefano et al., 2006; Svendsen, 1980). Generally, male territory owners have more tail wounds than females (Mayer et al., 2020; Müller‐Schwarze & Schulte, 1999), probably because they invest more time in territorial behaviors (Rosell & Thomsen, 2006; Sharpe & Rosell, 2003). There is no evidence for intragroup aggression in beavers (Baker & Hill, 2003; Mott et al., 2011). Consequently, kits have fewer injuries than adults, but yearlings and subordinates do not (Crawford et al., 2015), potentially because they risk physical disputes during dispersal attempts to obtain a territory (Mayer, et al., 2017; Tinnesand et al., 2013). Population density can also affect aggression in beavers. Mayer et al. (2020) demonstrated that territory owners have more tail scars at lower population densities due to increased intruder pressure via dispersers, because dispersers are more likely to emigrate at lower population densities and remain in their natal family group at high densities when the chances of obtaining a territory are low (Mayer, et al., 2017; Mayer Zedrosser et al., 2017c). Moreover, landscape structure can influence intraspecific aggression, with beavers in large rivers having more injuries compared to ones in smaller streams (Crawford et al., 2015). As intraspecific aggression is an important mechanism of mate change (Mayer, et al., 2017), and consequently territory occupancy and reproductive success (Mayer et al., 2020; Mayer, et al., 2017; Mayer Zedrosser et al., 2017a), it is important to understand the drivers of this behavior.

Here, we used long‐term data from Eurasian and North American beavers to investigate the effects of population density and landscape structure (here expressed as water body type) on the number of tail scars, our measure for intraspecific aggression. For well‐connected river systems and large lakes, we predicted that intraspecific aggression first increases with population density (in nonsaturated populations) due to increasing intruder pressure by dispersers, but then decreases at very high densities (in saturated populations) via a reduced intruder pressure because subordinates await lower densities for dispersal. Conversely, increasing population density might increase aggression in more isolated territories, located in smaller lakes and ponds, because explorative forays are more costly, forcing dispersers to attempt acquiring a territory rather than returning to the natal territory. Additionally, we investigated if the number of tail scars was related to sex, age, and (for Eurasian beavers only) social status and group size. We predicted that males have more scars than females due to increased investment in territorial behaviors, and similarly that territory owners have more scars compared to subordinates and kits. Moreover, we predicted that the number of tail scars increases with age, because scars accumulate over time, and that group size does not affect the number of tail scars, if there is no intragroup aggression in beavers.

## 2 MATERIAL AND METHODS

### 2.1 Study areas and data collection

#### 2.1.1 Eurasian beavers

**FIGURE 2:**
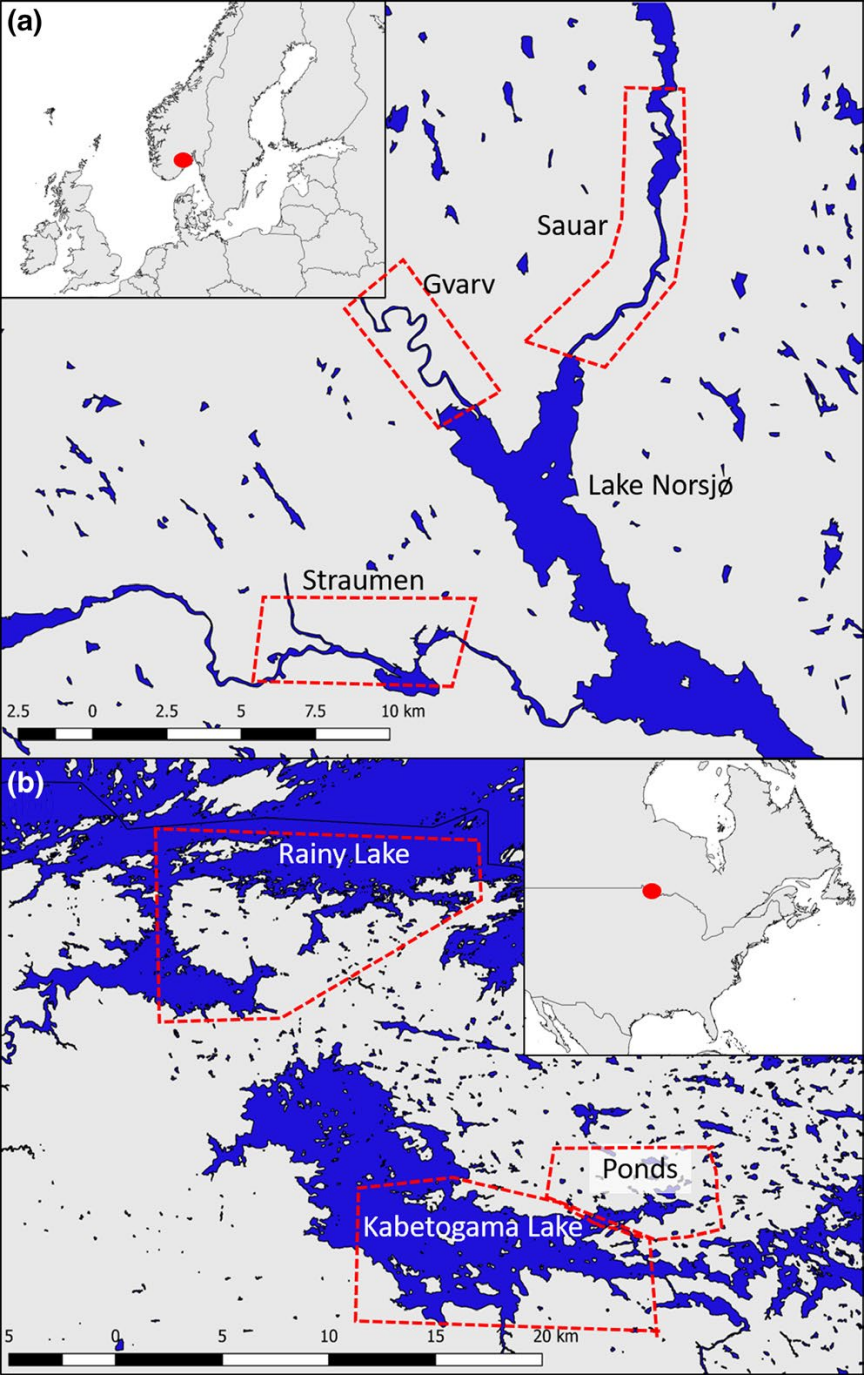
Maps showing our study areas (red dot in small maps shows location) in Norway (a) and the USA (b). Blue represents water bodies, and study areas are shown with red dashed lines

The study area in Vestfold and Telemark county, south‐eastern Norway, consisted of the rivers Sauar (59°444′N, 09°307′E), Gvarv (59°386′N, 09°179′E), and Straumen (59°297′N, 09°153′E), which are all interconnected by Lake Norsjø (Figure 2). The rivers are between 30 and 150 m wide and represent comparable landscapes. There is only localized ice cover in winter with most areas being ice‐free throughout the year. The landscape is semi‐agricultural with riparian woodland structures (Haarberg & Rosell, 2006). Beavers do not build dams in the rivers, as riverine habitats are naturally deep and wide enough (Hartman & Törnlöv, 2006). Hunting pressure was low to moderate (Herfindal et al., 2005; Mayer, 2017). Lynx (*Lynx lynx*) and red fox (*Vulpes vulpes*) were the only potential predators present in the area, suggesting a low natural predation pressure (Rosell & Sanda, 2006).

**FIGURE 3:**
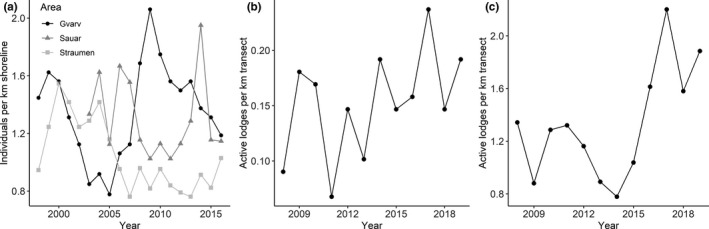
Variation in population density over time for the Eurasian beaver population (separately by river; a) and the North American beaver population, separately for large lakes (b) and ponds (c). Our intensive capture–mark recapture study in Norway allowed us to estimate population density as individuals per km shoreline. In North America, we used aerial surveys to estimate population density, given as active lodges per km transect. Large lakes and ponds for the North American site are given in different plots, because relative densities varied considerably

Eurasian beavers have inhabited the area since at least the 1920s (Olstad, 1937). The population has been at carrying capacity for the last 15 years with territories directly adjacent to each other and no unoccupied areas (Campbell et al., 2005; Mayer et al., 2020). Nevertheless, population densities varied over time via varying reproductive output (Figure 3). Data were collected every year from 1998 to 2016 as part of a long‐term individual‐based study. Beavers were captured at night during spring (March–June) and fall (August–November) from a motor boat using a landing net (Rosell & Hovde, 2001). Captured animals were individually marked and sexed based on the color and viscosity of the anal gland secretion (Rosell & Sun, 1999) and assigned to a social status (territory owner, subordinate, yearling, kit) (Mayer, et al., 2017; Mayer Zedrosser et al., 2017c). We counted the family group size for every territory in our study area using a combination of live captures and observations, allowing us to calculate the local population density (hereafter population density) separately for each river and each year (Figure 3), defined as the number of individuals per km shoreline (calculated for both sides of the river) (Mayer et al., 2020).We recorded tail scars every time we (re)captured an individual.

#### 2.1.2 North American beavers

The study area in Voyageurs National Park, Minnesota, USA (48°36′N, 93°25′W; 88,628 ha), is comprised of a matrix of large, interconnected lakes and 1,000s of beaver‐created impoundments within contiguous southern boreal forest (Figure 2) (Kallemeyn et al., 2003). Beavers in the large lakes do not build dams, but frequently dam small streams and wetlands to create ponds (Johnston & Windels, 2015). Generally, ice‐in occurs in mid‐November and ice‐out in late April or early May (Kallemeyn et al., 2003). Beaver harvest has been prohibited within the boundaries of the park since 1975, but fur trapping is legal and widespread in the surrounding areas. The main predator of beavers are gray wolves, which are abundant in the study area (Gable & Windels, 2018), and other occasional predators such as black bears (*Ursus americanus*), bobcat (*Lynx rufus*), Canada lynx (*Lynx canadensis*), and coyote (*Canis latrans*) are also present.

As part of a long‐term research program, beavers were live‐trapped in September‐October (2008–2019) and May (2009–2010) in three areas: Rainy Lake and Kabetogama Lake, which are both large lakes, and an area mostly consisting of ponds (Figure 2). Beavers were live‐trapped using Hancock‐style live traps set near active lodges and checked daily (Windels, 2014). Each beaver was ear‐tagged, weighed (±0.1 kg), and sexed based on the presence of an externally palpated baculum (Osborn, 1955), genetic analysis (Williams et al., 2004), or necropsy of recovered dead beavers. Individuals were classified as kit (<1 year old), “subadult” (1–3 year old), or adult (>3 year old; Windels, 2014) based on trapping history, sectioned teeth of recovered dead beavers, or based on morphometric measurements. Trapping methods differed from those in Norway in that we could not reliably determine social status of individuals. Finally, we estimated population density each year based on a long‐term aerial survey protocol by counting active beaver lodges (Johnston & Windels, 2015), separately for large lakes (Kabetogama and Rainy Lake) and ponds. From this, we calculated the number of active lodges (i.e., family groups) per km transect (Figure 3). We did not have information regarding family group sizes and thus could not use the same population density estimate as for Eurasian beavers.

### 2.2 Tail scars

**FIGURE 4:**
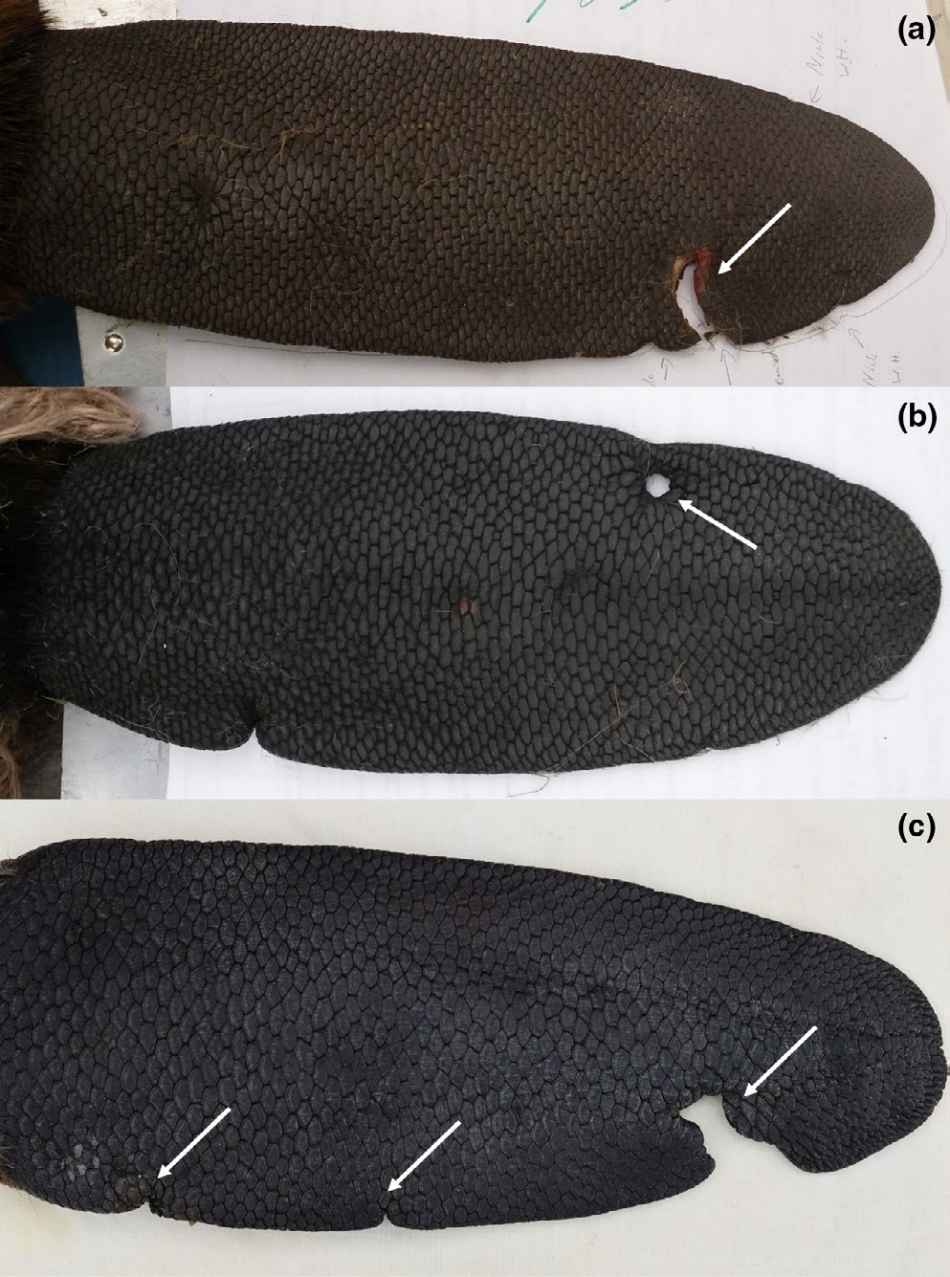
Example of a fresh tail scar caused by a conspecific (a) and a healed scar caused by a wolf (b) in North American beavers (*Castor canadensis*), and healed tail scars (c) caused by conspecifics in a Eurasian beaver (*Castor fiber*). Scars are indicated with white arrows. Pictures: Steve K. Windels (a, b) and Frank Rosell (c)

For each beaver, we recorded tail scars (Figure 4). In both study areas, the most common scars, termed “nicks,” were ignored for all subsequent analyses as they were very small (<0.6 mm^2^) and it was difficult to determine if they were congenital deformities along the margin of the tail or caused by injury. We also excluded scars caused by unnatural means, that is, those caused by traps or by placement of tail‐mounted transmitters (Windels & Belant, 2016). Based on tooth imprints and scar shape, we could usually distinguish between scars from conspecifics (crescent‐shaped injuries and cuts) and predators (point‐shaped injuries; only observed in the US; Figure 4). As scars were visible for long periods (years), we counted the cumulative number of tail scars over time.

### 2.3 Statistical analysis

We analyzed the number of tail scars (dependent variable in all analyses; Table 1) in a given year (each time an individual was captured) using generalized linear mixed models (GLMM) with a log link and a negative binomial distribution to account for overdispersion and zero inflation of the count data (Ver Hoef & Boveng, 2007) using the R package glmmTMB (Brooks et al., 2017; Magnusson et al., 2017). We initially analyzed all data combined and included the species, age category (kit, subadult (1–3 yrs. old), adult), sex, and the interactions of species × age category and species × sex as fixed effects and individual ID as random intercept to account for multiple observations. We then analyzed the data separately for Eurasian and North American beavers to include fixed effects that were calculated or categorized differently between the two study areas. For the analyses of Eurasian beavers, we included group size, the quadratic function of population density (fitted better than the linear effect; ∆ Akaike Information Criterion (AIC) = 13.4), social status (kit, yearling, subordinate, territory owner), sex, and biologically relevant two‐way interactions (Table 1) as fixed effects and individual ID as random intercept. Age was modeled separately for territory owners and subordinates (using the same model structure and variables as above), because age and social status were highly correlated (Spearman *r* > 0.6). For the North American data, we included the age category (kit, subadult, adult), area (Rainy Lake, Kabetogama Lake, ponds), sex, and the interactions of age category × area, and sex × age category as fixed effects and individual ID as random intercept. Body mass, as proxy of age (Mayer, et al., 2017), was correlated with the age category and was thus analyzed separately for adults and subadults. Population densities varied an order of magnitude between large lakes and ponds due to landscape composition (i.e., beavers in large lakes can only occupy lodges along the 1‐dimensional shoreline, whereas ponds can occur in the two dimensional matrix of forest uplands). We thus analyzed lakes and ponds in separate analyses, including the linear function of population density (active lodges per km transect; fitted better than the quadratic function), age category, sex, and biologically relevant interactions (no interactions were included in the “ponds” analysis to avoid overfitting the models; see results and Table 1) as fixed effects and individual ID as random intercept. There was no collinearity (Pearson *r* < 0.6 and variance inflation factors < 3) between fixed effects within the same model in any analysis (Zuur et al., 2010). We could not analyze the occurrence of new tail scars in a given year, because we had too few re‐captures of individual beavers to reliably quantify this measure. Model selection for all analyses was based on stepwise variable selection using AIC corrected for small sample size (AIC_c_), selecting the model with the lowest AIC_c_ (Murtaugh, 2009), using the R package “MuMIn” (Barton, 2016). If two or more models were within ∆AIC_c_ < 2, we selected the model with fewer parameters. Parameters that included zero within their 95% CI were considered uninformative (Arnold, 2010). We validated the most parsimonious models by plotting the model residuals versus the fitted values (Zuur et al., 2010). All statistical analyses were carried out in R 3.6.0 (R Core Team, 2013).

**TABLE 1:** Overview of the best and full model for all analyses conducted for the number of tail scars in Eurasian and North American beavers

Analysis/Model	Fixed effects	Link	Zero‐inflated	*df*	logLik	AICc	deltaAIC	AIC weight	R^2^m	R^2^c
Number of tail scars in Eurasian and North American beavers										
Best	Species + Age category	log (negative binomial)	Yes	7	−1,972	3,958	0	0.71	0.43	0.73
Full	Species + Age category + Sex + Species × Age category + Species × Sex	log (negative binomial)	Yes	11	−1,969	3,960	2	0.29	0.47	0.75
Number of tail scars in Eurasian beavers										
Best	Population density + Population density^2^ + Social status + Sex + Sex × Social status	log (normal)	No	11	−1,064	2,150	0	0.997	0.49	0.76
Full	Group size + Population density + Population density^2^ + Social status + Sex + Sex × Population density + Sex × Social status + Social status × Group size + Social status × Population density	log (normal)	No	19	−1,061	2,162	11.75	0.003	0.50	0.76
Number of tail scars in North American beavers (all data)										
Best	Age category + Area	log (negative binomial)	Yes	8	−869	1,755	0	0.88	0.38	0.72
Full	Age category + Sex + Area + Age category × Sex	log (negative binomial)	Yes	11	−868	1,759	4	0.12	0.39	0.72
Number of tail scars in North American beavers (Lakes)										
Best	Age category + Sex + Population density + Lake (Kabetogama vs. Rainy)	log (negative binomial)	Yes	8	−805	1,626	0	1.00	0.40	0.72
Full	Age category + Sex + Population density + Lake (Kabetogama vs. Rainy) + Age category × Sex + Age category × Population density + Population density × Sex	log (negative binomial)	Yes	13	−807	1,640	14	0.00	0.42	0.73
Number of tail scars in North American beavers (Ponds)										
Best	Age category	log (negative binomial)	Yes	5	−61	132	0	0.88	0.18	0.64
Full	Age category + Sex + Population density	log (negative binomial)	Yes	7	−61	136	4	0.12	0.19	0.65

Note

R^2^m = marginal R^2^ (fixed effects only), R^2^c = conditional R^2^ (fixed effects and random intercept).

## 3 RESULTS

### 3.1 Comparing Eurasian and North American beavers

**FIGURE 5:**
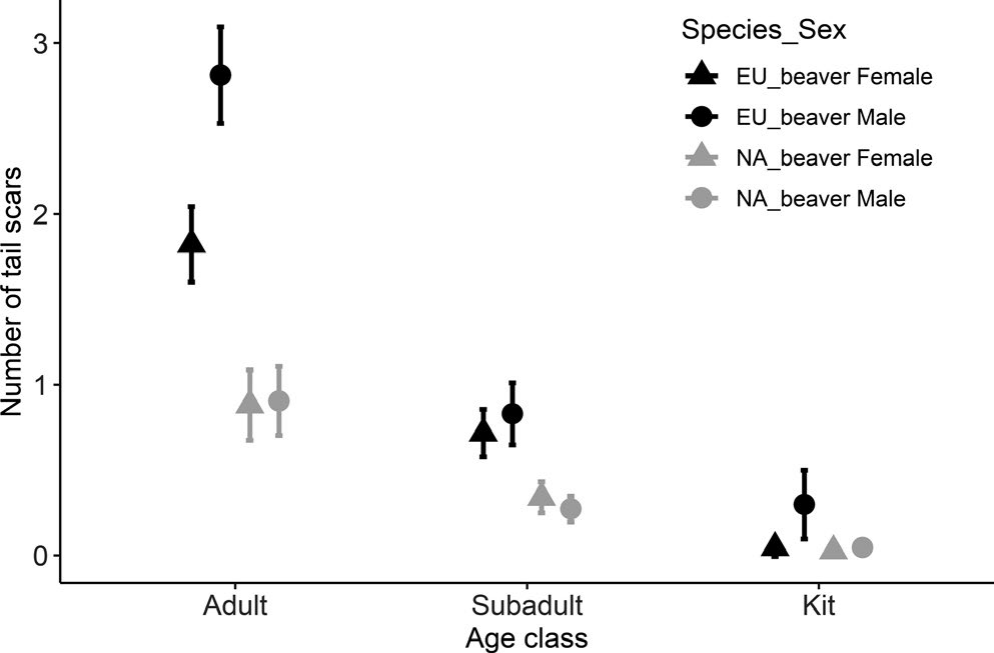
The mean number of tail scars (± 95% confidence interval) separately for females (triangles) and males (circles) shown for the different age categories and for the two beaver species (Eurasian beaver = black, North American beaver = gray)

We captured 335 Eurasian beavers (163 females and 172 males) from 1–13 times (mean ± *SD*: 2.6 ± 2.2), leading to a combined sample of 860 tail observations. We obtained 409 tail observations from territory owners, 187 from subordinates, 122 from yearlings, and 142 from kits (366 adults, 352 subadults and 142 kits when using the age categorization for North American beavers). Further, we captured 873 North American beavers (437 males and 448 females) from 1–5 times (mean ± *SD*: 1.5 ± 0.7), for a combined 1,278 observations (368 adults, 518 subadults and 392 kits). Eurasian beavers had more tail scars compared to North American beavers, and adults had more scars than subadults or kits (Figure 5, Table 2). Sex and the interactions of species × sex and species × age category were not included in the final model and uninformative in the full model.

**TABLE 2:** Estimate, standard error (*SE*), lower 95% confidence interval (LCI), and upper 95% confidence interval (UCI) of explanatory variables for the analyses of the number of tail scars in Eurasian and North American beavers

Variable	Estimate	*SE*	LCI	UCI
Number of tail scars in Eurasian and North American beavers				
Intercept	**0.32**	**0.09**	**0.14**	**0.51**
Species North American beaver	**−1.06**	**0.11**	**−1.27**	**−0.85**
Age category Kit	**−2.74**	**0.18**	**−3.08**	**−2.39**
Age category Subadult	**−1.00**	**0.07**	**−1.15**	**−0.86**
Number of tail scars in Eurasian beavers				
Intercept	**−1.12**	**0.78**	**−2.65**	**0.40**
Population density	**−3.18**	**0.85**	**−4.84**	**−1.52**
Population density^2^	**1.04**	**0.32**	**0.42**	**1.66**
Sex Male	**1.70**	**0.62**	**0.49**	**2.91**
Social status Subordinate	**3.04**	**0.59**	**1.88**	**4.20**
Social status Territory owner	**3.53**	**0.58**	**2.39**	**4.67**
Social status Yearling	**2.16**	**0.61**	**0.96**	**3.35**
Sex Male × Social status Subordinate	**−1.56**	**0.65**	**−2.83**	**−0.30**
Sex Male × Social status Territory owner	**−1.26**	**0.64**	**−2.51**	**−0.01**
Sex Male × Social status Yearling	**−1.92**	**0.68**	**−3.25**	**−0.58**
Number of tail scars in North American beavers (all data)				
Intercept	**−0.93**	**0.19**	**−1.29**	**−0.56**
Age category Kit	**−3.17**	**0.30**	**−3.76**	**−2.57**
Age category Subadult	**−1.06**	**0.14**	**−1.33**	**−0.79**
Area category Ponds	−0.55	0.29	−1.11	0.01
Area category Rainy Lake	**0.46**	**0.16**	**0.15**	**0.77**
Number of tail scars in North American beavers (Lakes)				
Intercept	**−1.40**	**0.27**	**−1.92**	**−0.88**
Population density	**3.35**	**1.32**	**0.76**	**5.94**
Age category Kit	**−3.21**	**0.31**	**−3.82**	**−2.60**
Age category Subadult	**−1.10**	**0.14**	**−1.38**	**−0.82**
Area category Rainy Lake	**0.44**	**0.16**	**0.14**	**0.75**
Number of tail scars in North American beavers (Ponds)				
Intercept	**−1.96**	**0.58**	**−3.11**	**−0.82**
Age category Kit	**−2.26**	**1.12**	**−4.45**	**−0.06**
Age category Subadult	−0.62	0.60	−1.79	0.55

Note

Informative parameters (95% confidence interval does not overlap zero) are given in bold.

### 3.2 Eurasian beaver

**FIGURE 6:**
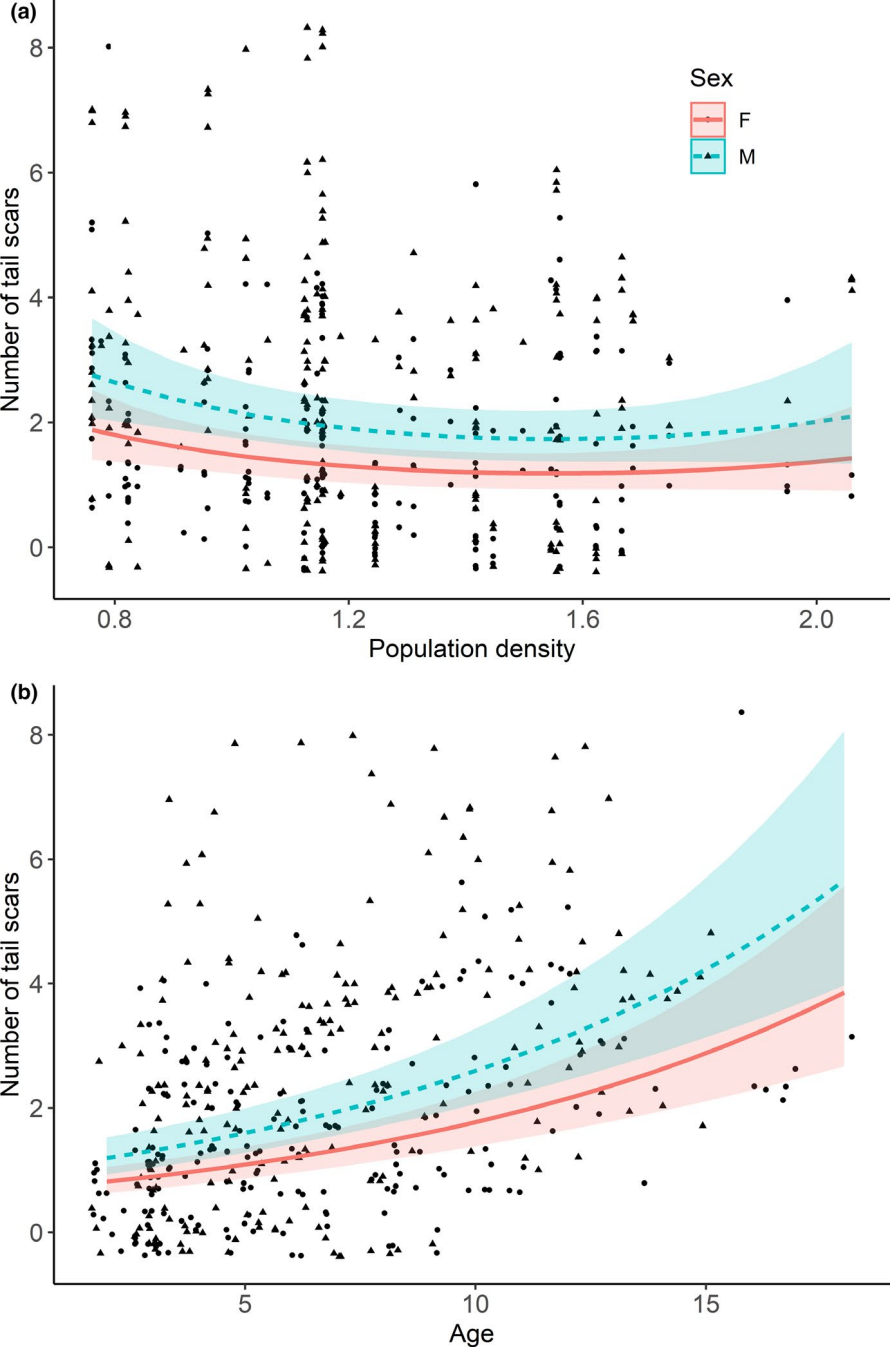
Showing the predicted effect (line) of population density (individuals per km shoreline; a) and age (b) on the number of tail scars in male and female territory owners (Eurasian beavers). Raw data are shown as dots (female) or triangles (male) and 95% confidence intervals are shown as shading

Eurasian beavers had between 0 and 8 tail scars (mean ± *SD*: 1.36 ± 1.96; median = 1). The quadratic function of population density revealed that the number of tail scars decreased with increasing population density, but then slightly increased at the highest population densities (Table 2; Figure 6). Moreover, the number of tail scars increased with an individuals’ age in subordinates (Estimate ± *SE*: 0.23 ± 0.05; not shown) and territory owners (Estimate ± *SE*: 0.10 ± 0.01; Figure 6). Territory owners had the most tail scars, followed by subordinates, yearlings, and kits (Table 2, Figure 5). Further, the interaction between social status × sex revealed that male territory owners had more scars compared to female territory owners (2.69 ± 1.67 vs. 1.65 ± 1.68), but this relationship was absent in subordinates and yearlings (Table 2; Figure 5). Surprisingly, male kits had more scars compared to female kits (0.30 ± 0.87 vs. 0.04 ± 0.21, Figure 5), but the median number of tail scars in both male and female kits was zero.

### 3.3 North American beaver

**FIGURE 7:**
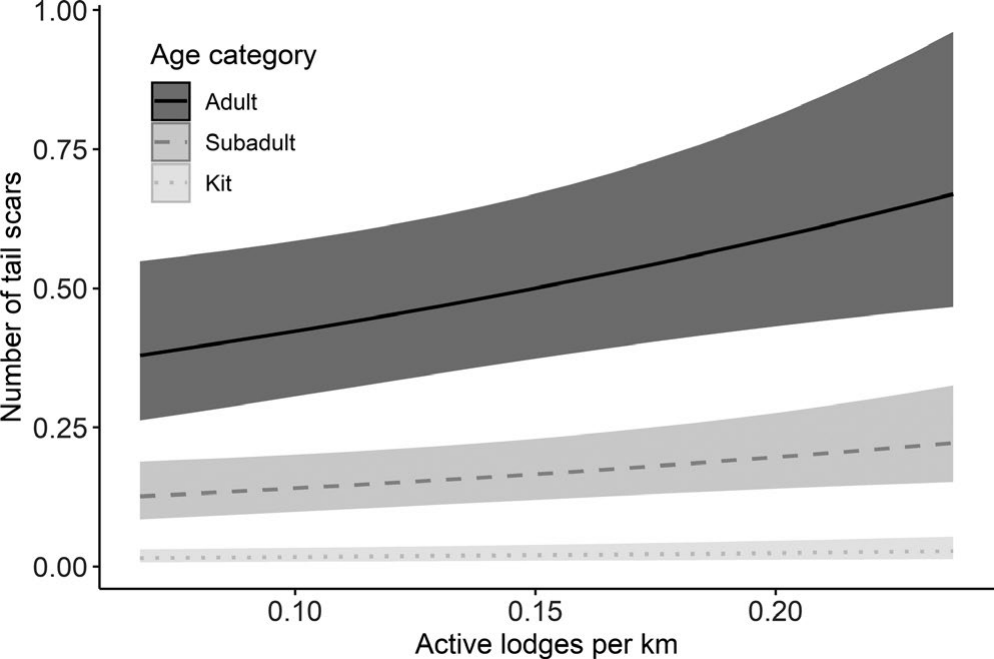
The effect of population density (measured as active lodges per km transect) on the number of tail scars of North American beavers in large lakes and separately for the age categories

The number of tail scars varied from 0–8 (mean ± *SD*: 0.39 ± 0.95; median = 0). Adults had the most scars, followed by subadults and kits (Table 2, Figure 5), and individuals generally had more scars in Rainy Lake (mean ± *SD*: 0.46 ± 1.03) than Kabetogama Lake (0.37 ± 0.92) and the ponds (0.21 ± 0.55, Table 2). Further, the number of tail scars increased with increasing body mass in adults (Estimate ± *SE*: 0.15 ± 0.03) and subadults (Estimate ± *SE*: 0.23 ± 0.04). Sex was uninformative in all models. When analyzed separately for large lakes (1,161 observations) and ponds (117 observations), we found that the number of tail scars increased with increasing population density in large lakes (Figure 7), but not in ponds (Table 1, 2). The population density estimate was almost 8‐fold higher in ponds compared to the large lakes (mean ± *SD*: 1.27 ± 0.52 vs. 0.16 ± 0.05 active lodges per km transect or 0.79 ± 0.32 vs. 0.10 ± 0.03 active lodges per km^2^; Figure 3).

## 4 DISCUSSION

We found that intraspecific aggression in both beaver species is common, caused by interterritorial conflicts, and depends on landscape structure and population density. Eurasian and North American beavers are strikingly similar regarding their biology (Rosell et al., 2005) and mostly differ genetically, having different karyotypes (Lavrov, 1973). Thus, differences in intraspecific aggression between the two studied populations were likely caused by differences in landscape structure and population density rather than biological species differences. In connected landscapes, such as large lakes and rivers, population density affected aggressive encounters, whereas this effect was absent in more isolated ponds. These findings provide empirical evidence to increase our general understanding of the role of intraspecific aggression on dispersal patterns (McCarthy, 1999), population dynamics (Cubaynes et al., 2014) and vice versa.

Territorial conflicts appear to be the primary driver of intraspecific aggression in beavers. Territory owners/adults and subordinates/subadults commonly had tail scars, whereas signs of intraspecific aggression were rare in yearlings and kits of both species. This is in line with previous studies (Crawford et al., 2015; Müller‐Schwarze & Schulte, 1999). If intragroup aggression in beavers exists, we would expect that kits and potentially yearlings have more tail scars with increasing group size. However, both the effect of group size and its interaction with social status were uninformative, suggesting that there are no or only minor intragroup disputes among beavers, in line with other studies (Baker & Hill, 2003; Crawford et al., 2015; Mott et al., 2011). We cannot exclude the possibility that subordinates compete with their parents for their natal territory, but have never observed any intrafamily group aggression during 24 years of intensive monitoring (FR, unpublished results). Conversely, subordinates in the Norwegian study population often delay dispersal and conduct extra‐territorial forays where they attempt to acquire a territory of their own (Mayer Zedrosser, & Rosell, 2017b, 2017c). This implies that the majority of tail scars resulted from interterritorial disputes, such as (attempted) territory acquisition by dispersing individuals and territory defense by territory owners, as shown in other mammals and birds (Jeschke et al., 2007; Lardy et al., 2011). However, it is plausible that subordinate siblings might compete for the same territory in some instances.

The Eurasian beaver population was saturated for most (if not all) of the study period with all territories adjacent to each other and no unoccupied areas (Campbell et al., 2005; Mayer, 2017). This was shown to cause inverse density‐dependent dispersal, that is, individuals delay natal dispersal to await comparatively lower population densities to increase their chances of obtaining a territory (Mayer et al., 2017; Mayer Zedrosser, & Rosell, 2017a, 2017c; Sun et al., 2000), also shown in other species (Ekman et al., 2001; Halliwell et al., 2017). In turn, this leads to high intruder pressure, and thus intraspecific aggression at lower densities (Mayer et al., 2020), and emphasizes that aggressive encounters might mediate natal dispersal decisions by individuals (Halliwell et al., 2017). The slight increase in intraspecific aggression at the highest population densities probably indicates that the sheer number of subordinates in the population led to increased intrusions or time spent in a transient dispersal stage (Maag et al., 2018), despite general inverse density‐dependent dispersal, causing more aggressive encounters. In contrast to the Eurasian population, the density in the North American population considerably increased toward the last years of this study (2015–2019), indicating that the population was in a growth phase and that not all areas were occupied by beaver territories during the earlier years of this study (2008–2014). This could potentially explain the positive relationship between intraspecific aggression and population density, if natal dispersal and consequent aggressive encounters were in a positive relationship with population density as shown in other species (Macdonald et al., 2004; Matthysen, 2005). However, preliminary data (SKW, unpublished results) suggest that, like in Eurasian beavers, natal dispersal was in an inverse density‐dependent relationship. More study is needed on dispersal rates and movement behavior in the North American population to better understand the drivers behind the patterns we describe here.

Importantly, the effect of population density was only apparent in the well‐connected larger lakes (North American beaver) and rivers (Eurasian beaver), allowing individuals to move relative freely between territories without physical barriers. Conversely, we found no effect of population density on intraspecific aggression in the less‐connected ponds where levels of aggression were generally lower, though these findings have to be taken cautiously due to the comparatively small sample size. In addition, population densities in ponds were much higher compared to large lakes, although it is hard to make a true comparison due to the fundamental differences in landscape composition. Importantly, population density varied largely over time in the ponds, making it likely that we would have detected density‐dependent effects should they have occurred. Taken together, these findings emphasize that intraspecific aggression does not only depend on population density, but to a large degree also on landscape composition. We speculate that lower levels of intraspecific aggression in ponds were caused by lower connectivity to other areas, in turn reducing the number of dispersing individuals reaching these areas. In line with this hypothesis, scent marking activity in North American beavers was shown to decrease with increasing distance to the next active territory (Müller‐Schwarze & Heckman, 1980). Moreover, it was shown in other species that dispersal, likely the main driver of intraspecific aggression in our system, and colonization success are highly dependent on landscape structure (Berggren et al., 2001), and that habitat structure affects the frequency of aggressive encounters (Kok et al., 2016).

Interestingly, male territory owners had more tail scars than females in Eurasian beavers, but not North American beavers. This pattern might be related to males seeking extra‐pair copulations, and we speculate that differences between the two populations were also caused by physical landscape differences during winter leading to varying ice cover. The study area in Norway remains ice‐free for most of the year and never completely freezes over, allowing individuals to move within the study area throughout the year (though movement can be constraint by cold water temperatures (Nolet & Rosell, 1994)). Conversely, the North American water bodies (both lakes and ponds) are covered by ice from December to April, preventing interterritorial movement. This period coincides with the beavers’ mating season, suggesting that mate guarding by male territory owners in the ice‐free Eurasian population and conflicts resulting from intruders that seek extra‐pair copulation lead to the observed sex differences. This hypothesis is partly supported by findings that extra‐pair copulations occur in the Norwegian population (Nimje et al., 2019), whereas no extra‐pair paternity was detected in a Russian population that experienced extensive ice cover during winter (Syrůčková et al., 2015). Moreover, Crawford et al. (2015) found no sex differences in conspecific aggression in areas that were also (at least in parts) covered by ice. Additionally, male territory owners invest more time in territorial behaviors in Eurasian beavers (Rosell & Thomsen, 2006; Sharpe & Rosell, 2003), potentially leading to more aggressive encounters as shown in badgers (*Meles meles*) (Macdonald et al., 2004).

Finally, the number of tail scars increased with age (or body mass in North America) in territory owners/adults and subordinates/subadults, indicating that scars accumulate over time. However, this effect was not entirely linear, suggesting that older individuals are more often involved in aggressive encounters. Previous studies in the Eurasian beaver population found that body mass of individuals declines (males) or stagnates (females) and that females have a decreased reproductive output in individuals ≥7 years old (Campbell et al., 2017; Mayer, et al., 2017), suggesting senescence in these individuals (18% of captures in this study). In turn, older territory owners showing signs of senescence might be challenged more often by dispersers, whose attempts at acquiring a territory likely also increase with age due to an enhanced competitive ability via increased body mass (Graf et al., 2016; Mayer, et al., 2017; Sun et al., 2000). Similarly, in badgers older and heavier individuals obtained comparatively more bite wounds (Macdonald et al., 2004).

Our results show that landscape structure in interaction with population density was an important driver of intraspecific aggression in beavers, which was likely mediated by dispersal patterns. In well‐connected landscapes, increased levels of intraspecific aggression, caused by positive (increasing population density) or inverse (saturated populations) density‐dependent dispersal, can lead to increased mate change and population turnover (Mayer et al., 2020), in turn potentially regulating population densities. In less‐connected landscapes, aggression appears to be density‐independent, suggesting that other factors than agonistic encounters via dispersers drive population dynamics there, such as resource availability (Fryxell, 2001). Our findings provide directions for future research. The question arises how landscape structure generally affects patterns of dispersal, mate change and territory occupancy, factors that will affect settlement patterns and reproductive success of individuals, and ultimately population dynamics (Berggren et al., 2001; Cubaynes et al., 2014). Moreover, future studies should aim to investigate the role of intraspecific aggression as mortality cause in animals, as it was shown that such mortality can be a substantial driver of population dynamics (Cubaynes et al., 2014). Answering those questions could aid reintroduction projects and more generally the management and conservation of wildlife species.

## CONFLICT OF INTEREST

The authors declare no conflict of interest.

## AUTHOR CONTRIBUTIONS

**Martin Mayer:** Conceptualization (equal); formal analysis (equal); methodology (equal); supervision (equal); visualization (equal); writing – original draft (lead). **Clàudia Aparicio Estalella:** conceptualization (equal); formal analysis (equal); writing – review and editing (equal). **Steve K. Windels:** conceptualization (equal); funding acquisition (equal); project administration (equal); resources (equal); writing – review and editing (equal). **Frank N. Rosell:** Conceptualization (equal); funding acquisition (equal); project administration (equal); resources (equal); writing – review and editing (equal).

## ETHICAL APPROVAL

All trapping and handling procedures in Norway were approved by the Norwegian Experimental Animal Board (id 742, id 2170) and the Norwegian Directorate for Nature Management (2008/14367 ART‐VI‐ID), which also granted permission to conduct fieldwork in our study area. Trapping and handling procedures in Voyageurs National Park were approved by U.S. National Park Service IACUC permit no.: MWR_VOYA_WINDELS_BEAVER. Our study met the ethics guidelines of the Association for the Study of Animal Behaviour (ASAB) (Buchanan et al., 2012).

## Data Availability

Relevant data are deposited in the online repository Dryad (Mayer, Martin (2020), TAIL_SCAR_DATA, Dryad, Dataset, https://doi.org/10.5061/dryad.qbzkh18g5).
